# The Impact of the Addition of Vitamins on a Silicone Lining Material to the Oral Mucosa Tissue—Evaluation of the Biocompatibility, Hydrolytic Stability and Histopathological Effect

**DOI:** 10.3390/medicina59111936

**Published:** 2023-11-01

**Authors:** Irina Gradinaru, Bianca Iulia Ciubotaru, Maria Butnaru, Florina Daniela Cojocaru, Costică Toader Covașă, Teofana Bibire, Mihaela Dascalu, Alexandra Bargan, Maria Cazacu, Mirela-Fernanda Zaltariov

**Affiliations:** 1 Department of Implantology, Removable Dentures, Technology, “Grigore T. Popa” University of Medicine and Pharmacy, 16 Universitatii Street, 700115 Iasi, Romania; irina.gradinaru@umfiasi.ro; 2 Department of Inorganic Polymers, “Petru Poni” Institute of Macromolecular Chemistry, Aleea Grigore Ghica Voda 41 A, 700487 Iasi, Romania; ciubotaru.bianca@icmpp.ro (B.I.C.); amihaela@icmpp.ro (M.D.); anistor@icmpp.ro (A.B.); mcazacu@icmpp.ro (M.C.); 3 Biomedical Sciences Department, Faculty of Medical Bioengineering, “Grigore T. Popa” University of Medicine and Pharmacy, 9-13 Kogalniceanu Street, 700454 Iasi, Romania; mariabutnaru@yahoo.com (M.B.); florinaivan90@yahoo.com (F.D.C.); 4 Faculty of Veterinary Medicine, “Ion Ionescu de la Brad” University of Life Sciences—IULS, Aleea Mihail Sadoveanu nr. 3, 700490 Iasi, Romania; costica_covasa@yahoo.com; 5 Faculty of General Medicine, “Grigore T. Popa” University of Medicine and Pharmacy, 16 Universitatii Street, 700115 Iasi, Romania; teofanabibire@yahoo.com

**Keywords:** lining materials, oral health, vitamins, biocompatibity, stability in biological media, histopathology

## Abstract

*Background and Objectives*: One’s quality of life depends on overall health, and in particular, oral health, which has been and continues to become a public health issue through frequent manifestations in various forms, from simple oral stomatitis (inflammations of the oral cavity) to the complicated oral health pathologies requiring medical interventions and treatments (caries, pulp necrosis and periodontitis). The aim of this study focused on the preparation and evaluation of vitamins (vitamin A, B1 and B6) incorporated into several silicone-based lining materials as a new alternative to therapeutically loaded materials designed as oral cavity lining materials in prosthodontics. *Materials and Methods*: Silicone-based liners containing vitamins were prepared by mixing them in solution and becoming crosslinked, and then they were characterized using Fourier-transform infrared (FT-IR) spectroscopy to confirm the incorporation of the vitamins into the silicone network; scanning electron microscopy (SEM) to evidence the morphology of the liner materials; dynamic vapor sorption (DVS) to evaluate their internal hydrophobicity, swelling in environments similar to biological fluids and mechanical test to demonstrate tensile strength; MTT to confirm their biocompatibility on normal cell cultures (fibroblast) and mucoadhesivity; and histopathological tests on porcine oral mucosa to highlight their potential utility as soft lining materials with improved efficiency. *Results*: FT-IR analysis confirmed the structural peculiarities of the prepared lining materials and the successful incorporation of vitamins into the silicone matrix. The surface roughness of the materials was lower than 0.2 μm, while in cross-section, the lining materials showed a compact morphology. It was found that the presence of vitamins induced a decrease in the main mechanical parameters (strength and elongation at break, Young’s modulus) and hydrophobicity, which varied from one vitamin to another. A swelling degree higher than 8% was found in PBS 6.8 (artificial saliva) and water. Hydrolytic stability studies in an artificial saliva medium showed the release of low concentrations of silicone and vitamin fragments in the first 24 h, which increased the swelling behavior of the materials, diffusion and solubility of the vitamins. The microscopic images of fibroblast cells incubated with vitamin liners revealed very good biocompatibility. Also, the silicone liners incorporating the vitamins showed good mucoadhesive properties. The appearance of some pathological disorders with autolysis processes was more pronounced in the case of vitamin A liners. *Conclusions*: The addition of the vitamins was shown to have a beneficial effect that was mainly manifested as increased biocompatibility, hydrolytic stability and mucoadhesiveness with the mucosa of the oral cavity and less of an effect on the mechanical strength. The obtained lining materials showed good resistance in simulated biological media but caused a pronounced autolysis phenomenon, as revealed by histopathological examination, showing that these materials may have broad implications in the treatment of oral diseases.

## 1. Introduction

Often, different types of lesions can occur in the oral mucosa while making an impression or extracting teeth from the oral cavity. Other injuries are due to patients’ pre-existing natural oral pathologies and mucosal diseases, which are not injuries caused by medical procedures or techniques. Like any procedure that aims to replace or bring new biomaterials into contact with oral tissues, the process of oral prosthetics represents a trauma to the patient. Regardless of the source of the lesion, all patients undergoing prosthetics need a rapid healing time of the oral tissues, not only for the success of the procedure but also for the overall well-being of the oral mucosa. The use of soft restorative materials can eliminate the risks associated with prosthetic injuries but cannot heal pre-existing ones [1].

The oral cavity has a limited surface area of around 50 cm^2^, but easy access to this site makes it a preferred site for drug administration. This site provides an opportunity to systemically deliver pharmacologically active agents by avoiding first-pass hepatic metabolism, in addition to the local treatment of pre-existing oral lesions, such as ulcerations, gingivitis, periodontal diseases or those produced during dental medical interventions [2,3].

Significant damage to supporting tissues during functioning can cause chronic pain, atrophic body changes and bone loss. Due to their elastic properties, soft lining materials are used to distribute functional and nonfunctional stresses more evenly and mitigate their effects. Soft denture lining materials are advantageous for treating patients with the following conditions: ridge atrophy or resorption, bony undercuts, bruxing tendencies, congenital or acquired oral defects requiring obturation, xerostomia and dentures that oppose the natural dentition in the opposite arch [4]. Failures are related to inadequate physical and mechanical qualities that cause bacterial and fungal growth on the lining materials and poor denture bonding [4]. Soft denture lining materials are commonly used in prosthodontics. Their softness and elasticity, which are qualities characterized by viscoelasticity, have been recognized as contributing to their success [5]. Periodontal disease has become a pathology problem involving the proximal tissues, causing tooth loss and requiring complex prosthetic treatments to avoid resorption of the jawbones, which is an ongoing process that often requires not only the lining of dentures but also their replacement. However, the body is capable of producing soft tissue after bone resorption, and thus, natural processes can intervene in the process of denture–prosthesis fixation, but do not provide stability, especially during mastication. Lining has become a specific procedure and is widely used in dentistry to design denture surfaces. The use of biodegradable and flexible acrylic materials is a good applied strategy with some short-term disadvantages related to the occurrence of oral infections caused by bacteria and fungi or material detachment. On the other hand, the use of hard acrylic materials reduces their tolerance by the patient, especially on the mucosal surface of the denture, while the elastic liners are resilient and can absorb energy and carry the cushion effect further. They are preferred by patients, but even their successful application depends on the surface properties of these materials, as the presence of roughness or defects will lead to microbial colonization. Another disadvantage is related to the stability of the liners in water, saliva and cleaning agents, which often induces hardness and physico-mechanical changes that affect their body safety [6,7]. 

Other causes are related to the external forces exhibited during mastication, which may lead to certain elastic deformations of the soft tissues. At this level, the mechanical properties are supported by the fiber content of the submucosa, and the effects are transmitted to the tissue–tooth interface [8]. The use of silicone-based dental soft lining materials and gels is a good alternative to acrylic-based materials. Silicones are biocompatible and possess good resistance to variable conditions and hygiene products used as denture cleaners, as well as offering good stability of shape and color [9] and they can be used as long-term liners, often up to one year [10].

The advantage of using silicone gel liners is that the liner distributes the pressure felt in a particular place, thereby reducing the pressure and increasing the ability to release the denture. The gels can also reduce friction with the oral mucosa that can occur during chewing food or speaking [11]. Soft liners also help patients tolerate their new mobile prosthesis by being fit exactly without damaging the soft tissue. Choosing the perfect material for such procedures must take into account several characteristics, in addition to good biocompatibility, “physical integrity”, good resilience and adhesion bond resistance, the latter of which can become a serious problem over time, leading to microbial colonization [12]. Silicone lining materials are usually marketed as paste/paste or paste/liquid with setting reaction conditions and fundamental composition aspects. Blends based on polyvinylsiloxane polymers produce stable composite materials without by-product removal and are available as a single-paste system with a free radical initiator. Investigation of these materials over three years showed that silicones remained stable, which recommends their use as permanent soft liners with higher durability and a lower damping effect, unlike acrylic materials [13].

The use of soft restorative materials significantly reduces the time needed to adapt to the new prosthesis and the interventions for necessary corrections. The most commonly used silicones (either as gels, pastes, liquid dispersions or elastomers) for long-term soft relining of removable dentures are Sofreliner Tough M and S (Tokuyama Dental Corporation, Tokyo, Japan), Mollosil Plus (DETAX, Ettlingen, Germany), Ufi Gel SC (VOCO, Cuxhaven, Germany), GC Reline Soft (GC Corporation, Tokyo, Japan), Elite Soft Relining (LavaDent, London, UK) and Molloplat B (DETAX, Ettlingen, Germany). What makes them recommended is the higher hardness stability over time compared with acrylic relining materials [14]. Long-term soft lining materials also have some disadvantages related to the loss of softness and water absorption, which mainly lead to microbial colonization and inflammation [15]. On the other hand, silicone materials have shown good resistance to disinfectants than acrylic materials [16].

Based on this motivation, in this study, we proposed to improve the effectiveness of silicone-based lining materials by incorporating bulking vitamins A, B1 and B6, which are involved in caries, periodontitis prevention and oral health [17]. Vitamin A refers to a family of hydrophobic substances that includes retinoids and carotenoids provitamin A. The retinol form, mostly in the *trans* configuration, has the highest vitamin activity. Nevertheless, the retinyl esters, mainly palmitate, stearate and palmitate, are the most prevalent forms of vitamin A present in tissues. The best-known use of this vitamin is with respect to vision. In addition, vitamin A affects the immune response [18]. Vitamin A was shown to prevent cleft palate. Deficiency of this vitamin can cause several dental abnormalities, brittle teeth, degeneration of salivary glands and dental caries. In many cases, periodontal diseases, anemia and a burning sensation in the oral cavity are associated with vitamin B6 deficiency [19]. Vitamin B1 is an indispensable nutrient for the central metabolism rooted in pyruvate, branched amino acids, and the 5-phosphate ribose and citric acid cycle [20]. It is well known that vitamins have a substantial impact on both oral and general health. There is a lack of literature on the contribution of vitamins to oral health, and there is no information on the incidence of oral diseases associated with vitamin deficiency. However, B vitamins were shown to have a large impact on the treatment of periodontal disease. A beneficial role of these vitamins in improving gingival inflammation in patients with poor oral hygiene was also observed. In these subjects, the long-term administration of vitamins A, B1, B6, C or D decreased the prevalence of gingivitis and mouth ulcers, including in diabetics [17]. In this study, we aimed to load these essential vitamins in silicone lining materials using an in situ crosslinking process of a silicone liner in the presence of vitamins by solvent casting method. The resulting materials were characterized using FT-IR spectroscopy to confirm the embedding of the vitamins in the silicone networks, dynamic vapor sorption (DVS analysis) to assess hydrophobicity and stability in a moist medium, a mechanical test to demonstrate tensile strength, MTT to confirm their biocompatibility on normal cell cultures (fibroblasts), and mucoadhesivity and histopathological tests on porcine oral mucosa to highlight their potential use as soft lining materials with improved efficiency.

## 2. Materials and Methods

### 2.1. Materials

Mollosil^®^ plus Primer (DETAX GmbH & Co. KG, Ettlingen, Germany), Mollosil Base and Catalyst (DETAX GmbH & Co. KG, Ettlingen, Germany), artificial saliva for pharmaceutical research (Aldrich, Darmstadt, Germany), Retinol (vitamin A) (Aldrich, Germany), Thiamine hydrochloride (vitamin B1—oil formulation) (Aldrich, Germany) and Pyridoxine hydrochloride (vitamin B6—oil formulation) (Aldrich, Germany) were purchased and used as received. Dulbecco’s Modified Eagle’s Medium/Nutrient Mixture F-12 Ham (Sigma-Aldrich, Steinheim, Germany); Trypsin from porcine pancreas, mol. wt. 23.8 kDa (Sigma-Aldrich, Steinheim, Germany); Penicillin-Streptomycin-Neomycin Solution—P/S/N, ~5000 units penicillin, 5 mg streptomycin and 10 mg neomycin/mL, 0.1 μm filtered (Sigma-Aldrich, Steinheim, Germany); Fetal Bovine Serum (FBS) (Sigma-Aldrich, Steinheim, Germany); Thiazolyl Blue Tetrazolium Bromide (MTT) 3-(4,5-Dimethyl-2-thiazolyl)-2,5-diphenyl-2H-tetrazolium bromide, Methylthiazolyldiphenyl-tetrazolium bromide (Sigma-Aldrich, Steinheim, Germany); Hanks’ Balanced Salt solution (HBSS), modified with sodium bicarbonate, without phenol red, calcium chloride and magnesium sulfate, liquid, sterile-filtered (Sigma-Aldrich, Germany); Formalin solution, neutral buffered 10% (Sigma-Aldrich, Germany); Mayer’s Hematoxylin solution (Sigma-Aldrich, Germany); Ammonia solution 2M in ethanol (Aldrich, Germany); Eosin (Aldrich, Germany); Dimethyl sulfoxide (DMSO) (Sigma-Aldrich, Germany); and Ethanol absolute (Chemical Company, Iasi, Romania) were used for the cell culture and hystopathological experiments.

### 2.2. Preparation of the Vitamin-Embedded Silicone-Based Lining Materials

The silicone lining materials were obtained via the in situ crosslinking of dental linings based on a catalyst and Mollosil base with different vitamins (A, B1 and B6) as oil formulations. Mixtures consisting of 0.25 g catalyst and 0.25 mL chloroform, and mixtures containing 0.25 g base and 0.005 g vitamin A dissolved in 0.25 mL of chloroform were stirred separately for 30 s, then combined and stirred together for another 30 s. The combined mixtures were poured onto a Teflon substrate and left overnight at room temperature to complete the crosslinking process (vitamin A liner). The prepared silicone liners were subsequently dried in a vacuum oven for 48 h at 25 °C. The other samples (the vitamin B1 and vitamin B6 liners) were designed according to the reagents shown in Table 1. A blank sample without vitamins was also prepared to compare the loading efficiencies of the loaded vitamins.

### 2.3. Methods

***ATR-FTIR*** spectra were recorded at room temperature on a Bruker Vertex 70 FTIR spectrometer equipped with a ZnSe crystal. Spectra were measured in the 4000–600 cm^−1^ range using 32 scans and a resolution of 4 cm^−1^. The spectra were processed in OPUS 6.5 software.

***Stress–strain curves*** were recorded on an Instron 3365 apparatus. The measurements were performed at room temperature with an extension rate of 200 mm/min. Young’s modulus was determined on the linear part of the stress–strain curves at 10% strain.

***The surface morphology*** of the silicone lining materials was investigated using a scanning electron microscope (Verios G4 UC, Thermo Scientific, Prague, Czech Republic). Samples were coated with 10 nm of platinum using a Leica EM ACE200 Sputter coater to ensure electrical conductivity and prevent charge accumulation during electron beam exposure. SEM investigations were performed in high-vacuum mode using a secondary electron detector (Everhart-Thornley detector) at an accelerating voltage of 5 kV.

***Dynamic vapor sorption (DVS)***: The behavior of the silicone liners in humidity environments was evaluated by measuring the dynamic water vapor sorption capacity using a fully automated gravimetric device IGAsorp manufactured by Hiden Analytical, Warrington, UK. This equipment possesses an ultrasensitive software-controlled microbalance, which allows the weight variation to be measured as the humidity changes at a predefined temperature. Before recording, samples were dried at 25 °C in flowing nitrogen (250 mL/min) to equilibrium. Then, the relative humidity (RH) was gradually increased from 0 to 90% in 10% humidity steps, with each sample having a pre-determined equilibrium time between 40 and 60 min, and the sorption equilibrium was obtained for each step. The RH was decreased and the desorption curves were registered.

By applying the Barrett, Joyner and Halenda model (BJF, Equations (1) and (2)), based on calculation methods for cylindrical pores, the average pore size $r_{pm}$ was estimated. This method uses the desorption branch of the isotherm. The amount of desorbed vapor is either due to the evaporation of the liquid core or the desorption of a multilayer. Pore size distribution is defined as the pore volume distribution. The relationship between pore volume and gas uptake can only be defined if the density of the adsorbed phase is known. The first assumption of mesopore size analysis is that this phase is equivalent to the liquid phase of the adsorbate.

(1) $$V_{liq}=\frac{n}{100\rho_{a}}$$

(2) $$r_{pm}=\frac{2V_{liq}}{A}$$

where $V_{liq}$ is the liquid volume, *n* is absorption percentage, $\rho_{a}$ is the adsorbed phase density and *A* is the specific surface area evaluated using the BET method.

To evaluate the specific surface area, the Brunauer–Emmett–Teller kinetic model (BET, Equation (3)) was applied by modeling the sorption isotherms registered under dynamic conditions.

(3) $$W=\frac{W_{m}CRH}{\left(1-RH\right)(1-RH+CRH)}$$

where the parameters involved are the weight of sorbed water—W, weight of the water forming a monolayer—$W_{m}$, sorption constant—*C* and relative humidity—*RH*.

***The swelling behavior*** of silicone-based lining materials was investigated in PBS pH 6.8 (simulating salivary fluid), water and formaldehyde (used in the sterilization process of lining materials). Before immersion in the chosen solutions, the weight of each sample was recorded. The silicone-based liners were placed into test vials containing 10 mL of each solution and incubated at 37 °C. The weight of the swollen liners was recorded at 24 h intervals for six days. The degree of swelling was determined using Equation (4):

(4) $$SD=\frac{M_{t}-M_{0}}{M_{0}}\times 100$$

where *SD* is the degree of swelling, while *M*_0_ and *M_t_* are the material mass (g) before and after immersing in solution for each 24 h interval, respectively.

***The hydrolytic stability*** of the silicone lining materials was evaluated in vitro in PBS phosphate buffer at pH 7.4 and in artificial saliva over 72 h. The biodegradation process was monitored by means of FT-IR spectroscopy using the subtraction function applied to IR spectra recorded before and after degradation every 24 h in both artificial saliva (at pH 6.8) and PBS (at pH 7.4). The difference between the spectra (subtraction function in OPUS 6.5 software) was used to confirm the presence of degradation by-products.

#### *In Vitro* Cytotoxicity Assay

The cytotoxicity of the silicone liners was evaluated in vitro on Albino rabbit dermal fibroblasts (RabDF, SCRab2300, ScienCell Research Laboratories, Carlsbad, CA, USA), split 10, isolated using the explants technique. The first step in performing the cytotoxicity assay was thawing the cells. The cryotube was removed from −86 °C, immediately immersed in water at 37 °C and gently shaken until completely thawed. The cells were centrifuged with a complete culture medium (DMEM with 10% FBS and 1% P/S/N) to remove the cryoprotectant (DMSO); then, the cells were counted using a Neubauer cell counting chamber. An appropriate volume of cell suspension was added to a culture flask (containing complete culture medium, equilibrated in the incubator 2 h before, at 37 °C and 5% CO_2_). The culture medium was replaced daily with a fresh one until a confluent monolayer was formed. The next step was to count the cell population of the culture plates (6-well plates). To detach the cells, the culture medium was removed from the flask, the surface was washed several times with HBSS to completely remove the culture medium and a volume of trypsin solution (0.025% trypsin and 0.02% EDTA) was added. After 1–2 min, the complete medium was added to stop the enzyme activity, then centrifuged and the supernatant was replaced with fresh medium. The Neubauer cell-counting camera was again used to count the cells, which were finally seeded at 6 × 10^4^ cells/well. 

**Figure 1:**
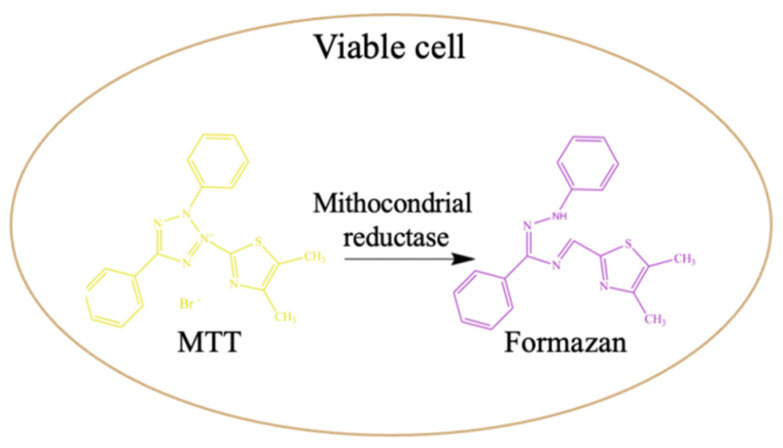
MTT assay principle.

To perform the MTT assay, the silicone liner aliquots were carefully removed from the wells using sterile tweezers, and the culture medium was replaced with an MTT working solution (5% MTT in DMEM). This assay is based on the ability of metabolically viable cells to reduce MTT in blue-violet formazan crystals (Figure 1) [21,22]. 

The cells with the MTT solution were incubated at 37 °C in 5% CO_2_ for 2 h, and then the MTT solution was removed and the formed formazan crystals were solubilized with DMSO. The absorbance of the resulting formazan solution (blue-violet) was measured at λ = 570 nm using a plate reader (Tecan Sun-rise Plate Reader, Tecan, Männedorf, Switzerland). The assay was performed at 24, 48 and 120 h.

The results from the experimental wells (*Abs material*) were compared with the control wells (*Abs control*) in which no film aliquots were present, resulting in the cell viability values *V* (%) (Equation(5)):

(5) $$V=\frac{Abs\;material\;}{Abs\;control}\times 100$$

***Bioadhesion and mucoadhesion tests*** were measured on TA.XTPlus^®^ texture equipment (Stable Micro Systems, Godalming, UK) by using a synthetic open-pore cellulose membrane obtained via boiling and step-cooling and a biologic mucosal tissue of porcine oral tissue. Prior to testing, all silicone lining materials were cut to a standard size (pieces of 4 cm^2^). For measurements, PBS pH 7.4 and artificial saliva (pH 6.8) were used to mimic physiological body conditions.Experiments were performed within the predefined software experimental settings: a holding instrument speed of 1 mm/s to reach the synthetic and biological mucosa, a contact force of 9.8 mN, and a contact time between the lining materials and biological mucosa or cellulose membrane of 30 s. The results obtained as detachment force and work of adhesion parameters were automatically calculated using TA.XT Plus^®^ texture equipment Exponent Connect software. The results from triplicate measurements were plotted with standard deviation.

***Histological analysis*** was performed on harvested oral mucosal biopsies, which were first fixed in 10% buffered formalin solution for 24 h, then sections were placed in hematoxylin for 2 min and in 0.5% ammonia for 1 min, and then washed and placed in eosin for 2 min using Thermo Scientific Microm HMS 70. The dehydration, cleaning, capping and paraffin embedding procedures were performed using a 120-3 Thermo Scientific STP tissue processor. Biopsy fragments were examined using an Olympus BX 41 microscope (Spectra Services, Ontario, NY, USA) coupled with an Olympus DP25 video camera.

## 3. Results

### 3.1. FT-IR Analysis

**Figure 2:**
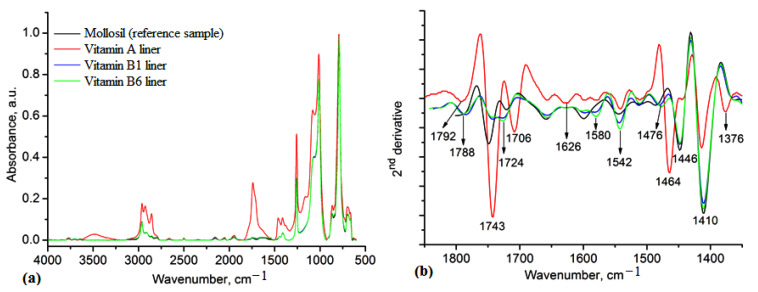
IR spectra of the silicone-based lining materials (**a**) and the second derivative of the spectra in the spectral range 1850–1350 cm^−1^ (**b**).

The structural features of the prepared lining materials were first investigated using IR spectroscopy. In all materials, the main absorption bands were attributed to siloxane networks and the Si-O-Si groups, which can be observed occurring at 1012 and 1080 cm^−1^, respectively. The stretching vibrations from 2962 to 2830 cm^−1^ are characteristic of asymmetric and symmetric stretching vibrations of C-H groups attached to silicon atoms. The corresponding deformations of these groups occurred at 1258 cm^−1^, while the rocking vibrations of Si-CH_3_ groups occurred at 789 cm^−1^ (Figure 2a). In the spectral region 1800–1350 cm^−1^, there were several overlapping bands attributed to the loaded vitamins. The maxima of these bands were determined using the second derivative of the spectra and the results are shown in Figure 2b. In the vitamin A liner, the maxima at 1743 cm^−1^, 1706 cm^−1^, 1464 cm^−1^ and 1376 cm^−1^ were specific to vitamin A, C-O and C=C conjugated groups and *cis* C-H double bonds, respectively [23], while in the vitamin B1 and B6 liners, maxima at 1580 cm^−1^, 1542 cm^−1^ and 1476 cm^−1^ were specific to C-N stretching and C-H deformation in vitamins B1 and B6 [24]. The pyridine ring in these vitamins appeared in the range of 1650–1300 cm^−1^ [25]. These vibrations confirm the embedding of vitamins in the matrix and the specific interactions with the silicone matrix. The changes in intensity and position of the initial bands in the reference sample spectrum, namely, 1728 cm^−1^, 1600 cm^−1^, 1446 cm^−1^, 1410 cm^−1^ and 1376 cm^−1^, compared with those containing vitamins, support the presence of vitamins. These were mainly attributed to the C-H rocking, twisting and wagging vibrations and confirm that some changes in the conformation of the silicone matrix occurred when the vitamins were added (Figure 2b). However, the most visible changes can be observed in the vitamin A liner spectrum due to the presence of multiple simple and double bonds in the vitamin A structure.

### 3.2. Mechanical Tests

**Figure 3:**
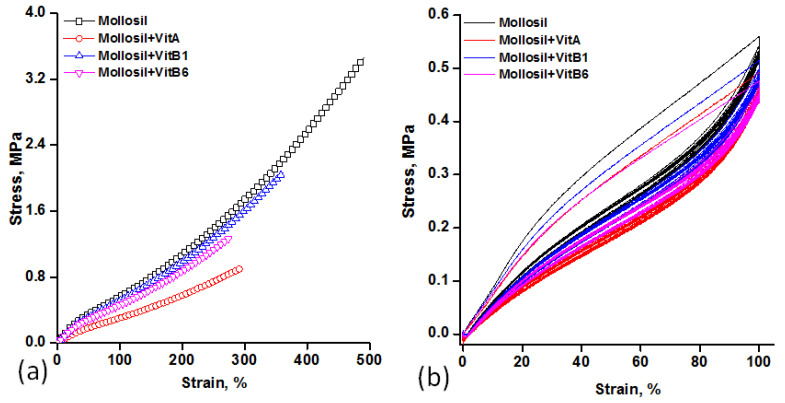
Stress–strain curves: linear (**a**) and cyclic (**b**) for the silicone-based vitamin-containing lining materials.

The results of the mechanical tests of the silicone-based lining materials are shown in Table 2 and Figure 3.

As can be seen, the incorporation of vitamins affected the mechanical properties of silicone, which led to a slight deterioration. Thus, the breaking strength and Young’s modulus decreased the most, by 73.4% and 54.6%, respectively, in the case of the vitamin A liner, while elongation decreased the most, by 43.3%, in the case of the vitamin B6 liner. The smallest changes in all mechanical characteristics were recorded for the vitamin B1 liner: 26.3% for elongation at break, 40.4% for breaking strength and 10.5% for Young’s modulus. These changes can be influenced by the degree of dispersion of the vitamin in the silicone matrix and by the size and shape of their aggregates.

**Figure 4:**
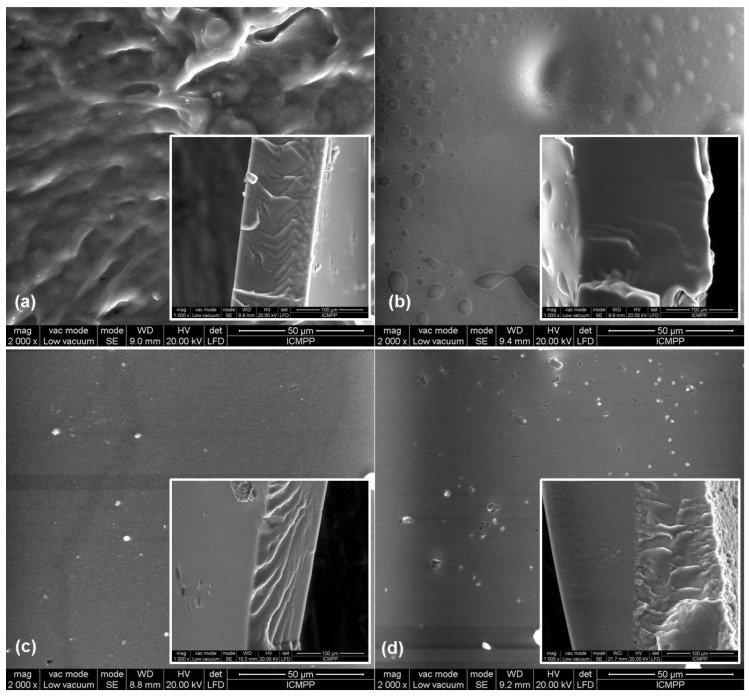
SEM images for vitamin-containing lining materials: (**a**) blank sample, (**b**) vitamin A liner, (**c**) vitamin B1 liner and (**d**) vitamin B6 liner. Inset graphs—SEM images of each silicone liner cross-section.

It was assumed and supported by SEM images (see Figure 4) that vitamin A1, with its flexible and amphiphilic, surfactant-like structure in the strongly non-polar environment created by the silicone matrix, forms well-defined aggregates whose presence worsens the mechanical properties due to weak interactions with the matrix. On the other hand, in the case of vitamin B1 with a more rigid structure, aggregation is probably less likely, which somewhat diminishes its effect on the mechanical properties of the silicone. 

The stress–strain cycles, which were performed at 100% elongation of the initial length (Figure 3b), revealed an elastic behavior, with the presence of a clearly visible hysteresis loop in all cases only for the first elongation–relaxation cycle, which did not disappear in the following cycles. The hysteresis loop is a measure of the energy dissipated due to internal friction.

### 3.3. Surface Analysis

#### 3.3.1. Morphology

Vitamins B1 and B6 are strongly hydrophilic, while the structure of vitamin A, which consists of a lipophilic permethylated organic fragment and a hydroxyl tail, is similar to a surfactant. When incorporating them into the hydrophobic silicone matrix, phase separation can occur, leading to the vitamins forming aggregates, both in the mass and on the surface. SEM images (Figure 4) revealed a compact morphology of the lining materials. In the case of the vitamin A liner, the surfactant character of vitamin A was reflected in the spherical shape of the aggregates, which are visible in particular on the surface (Figure 4b). In the cross-section, the lining materials exhibited a compact morphology, except for the blank sample (Figure 4a) and vitamin B6 liner (Figure 4d), where the structuring of the materials with the formation of channels can be observed. These could even be created via phase separation, or during the crosslinking process, as in the case of the blank sample. In this case, it is possible that these channels are created via dehydrocoupling reactions with hydrogen release, which competes under certain conditions with the hydrosilylation reaction used for the crosslinking process.

#### 3.3.2. Dynamic Water Vapor Sorption Analysis

The water vapors sorption capacity in the dynamic regime of the vitamin-incorporated silicone materials was evaluated and depended mainly on the porosity of the sample and the hydrophilicity of the internal surface. The obtained values (Table 3) revealed hydrophobic materials, with the lowest values being recorded for the reference sample and the vitamin B6 liner (0.65%), while a higher value was observed for the vitamin B1 liner (4.8%). Table 3 shows the surface parameters resulting from the sorption and desorption isotherms results.

The BET model describes the sorption isotherms up to a relative humidity of 40%, depending on the type of sorption isotherm and the type of material. This method can describe the isotherms of types II, as well as types I, III and IV. The average pore sizes also influenced, in a complex manner, the sorption capacity of the studied samples, with these being more hydrophobic.

### 3.4. Swelling Behavior

**Figure 5:**
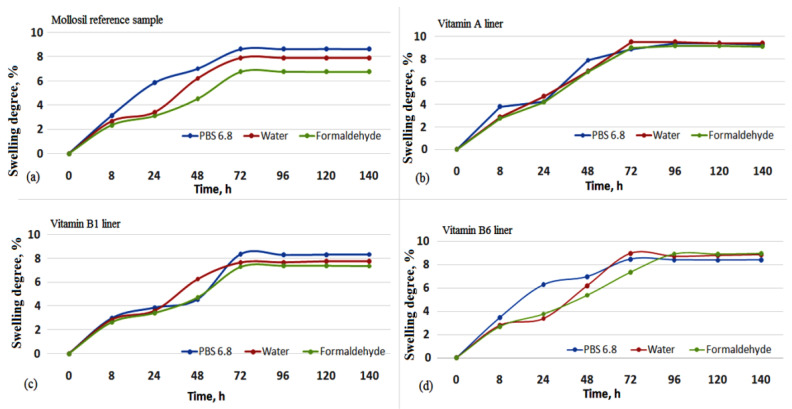
The swelling degree of the silicone lining materials: (**a**) the reference sample, (**b**) the vitamin A-based liner, (**c**) the vitamin B1-based liner and (**d**) the vitamin B6-based liner in different media: PBS 6.8 (artificial saliva), water and formaldehyde.

The degree of swelling of the vitamin-containing silicone liners was monitored for 140 h in various environments and conditions that are encountered during the handling and or storage of these materials (Figure 5). 

After analyzing the degree of swelling in the first 24 h, it can be seen that PBS 6.8 medium (artificial saliva) caused a higher degree of swelling of the reference lining material and vitamin B6 liner (6%), with lower values being found for the vitamin A liner and the vitamin B1 liner (4%), but higher values in water, than the other two, similar to other literature reports [26]. The chemical composition of artificial saliva, like biological saliva, is responsible for multiple functions: the dilution or removal of acid concentration, minimizing the pH drop, providing an ionic environment that speeds up healing in diseases of the oral cavity and preventing demineralization of enamel to acid attack during food intake [27]. The swelling behavior of silicone-based lining materials in artificial saliva was influenced by the pH and electrolyte concentrations. Phosphate ions in the composition of artificial saliva and the presence of the hydrosoluble vitamin B6 in the lining materials also caused a higher degree of swelling, while the swelling of the Mollosil reference sample can be explained by the catalytic hydrolytic effect of phosphate ions [28].

After 72 h, all lining materials showed a swelling degree greater than 8% in PBS 6.8 and water, followed by equilibration at a longer time.

### 3.5. Hydrolytic Stability

The stability of silicone-based lining materials was studied in PBS 7.4 and artificial saliva (PBS 6.8) for 72 h because, as shown in the swelling data, these materials sustained different degrees of swelling and it is important to determine whether these media can cause hydrolysis by-products. 

**Figure 6:**
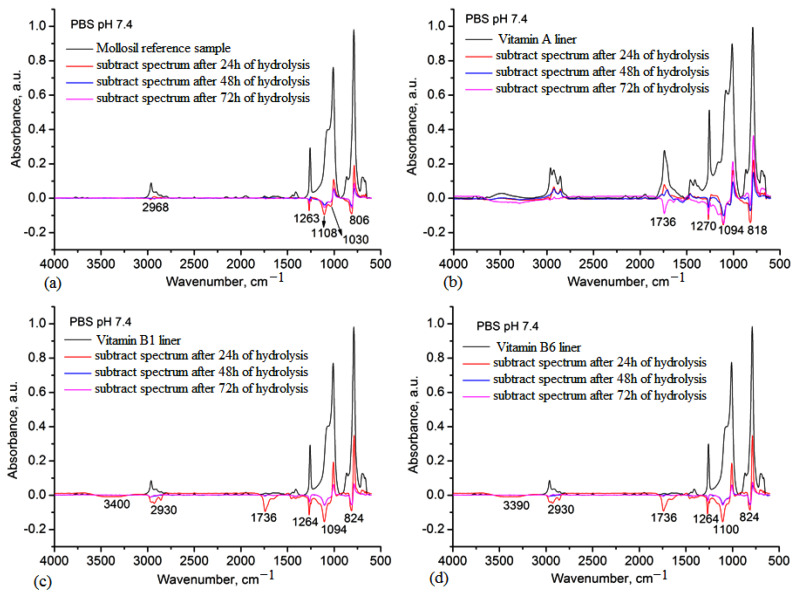
IR subtraction spectra of the silicone-based lining materials immersed in PBS 7.4 over 72 h: (**a**) the Mollosil reference sample, (**b**) the vitamin A liner, (**c**) the vitamin B1 liner and (**d**) the vitamin B6 liner. The marked bands were assigned to the silicone or vitamin fragments released in the medium as an effect of swelling and hydrolytic degradation.

Using the IR subtracting procedure, the main spectral changes can be observed after immersion in these media. Thus, the studied IR difference spectra between the initial spectra and those that resulted after immersion for 24 h, 48 h and 72 h are shown in Figure 6. In all sample cases, after 24 h, silicone fragments from the matrix appeared in the medium; this was more pronounced for the blank reference sample (Figure 6a) and the vitamin B1 liner (Figure 6c). This process was continuous for the reference sample over the full 72 h, while for the samples containing vitamins, these fragments occurred only in the first 24 h. For the vitamin A liner, the main products consisting of vitamin A appeared after 72 h, while for the vitamin B1 liner and the vitamin B6 liner, the vitamin fragments appeared after 48 h. This behavior can be explained by the influence of the phosphate buffer on the silicone matrix and the diffusion of the vitamin fragments in the medium [29]. However, the release of the vitamins is beneficial in this case, as these vitamins have therapeutic effects on the oral mucosa [30].

**Figure 7:**
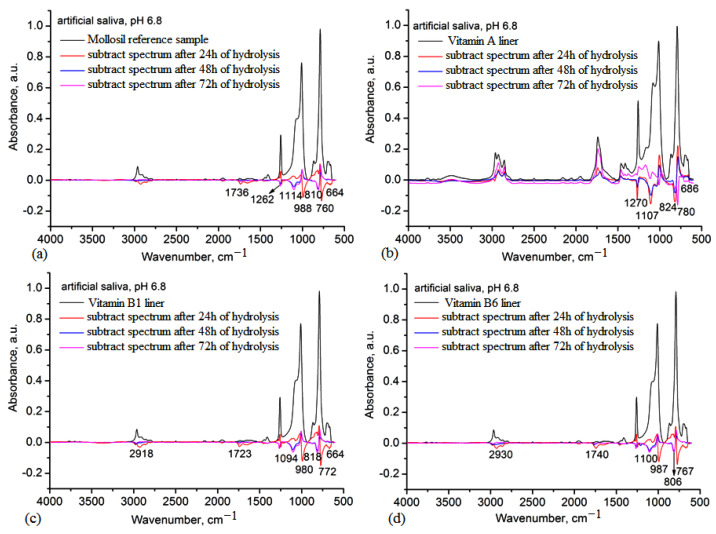
IR subtraction spectra of the silicone-based lining materials immersed in artificial saliva over 72 h: (**a**) the Mollosil reference sample, (**b**) the vitamin A liner, (**c**) the vitamin B1 liner and (**d**) the vitamin B6 liner. The marked bands were assigned to the silicone or vitamin fragments released in the medium as an effect of swelling and hydrolytic degradation.

The analysis of the hydrolytic stability in artificial saliva medium revealed a similar behavior to in the PBS 7.4 medium, with the release of small silicone fragments in all samples being more pronounced for the reference and vitamin A liner (Figure 7a,b), while for the vitamin B1 and vitamin B6 liners, this process occurred only in the first 24 h (Figure 7c,d). The appearance of vitamin fragments in the salivary medium was also caused by the swelling behavior of the samples in saliva, diffusion and solubility of vitamins in saliva. Based on these data, higher stability was observed for the vitamin B1 liner, even though this material also showed a slightly higher water sorption capacity and a higher degree of swelling in PBS 6.8 after 72 h. 

### 3.6. In Vitro Cytotoxicity Assay

The results of the cytotoxicity assay are shown in Table 4. As the values were higher than 95%, even after 120 h of direct contact, the films can be considered non-cytotoxic in vitro.

**Figure 8:**
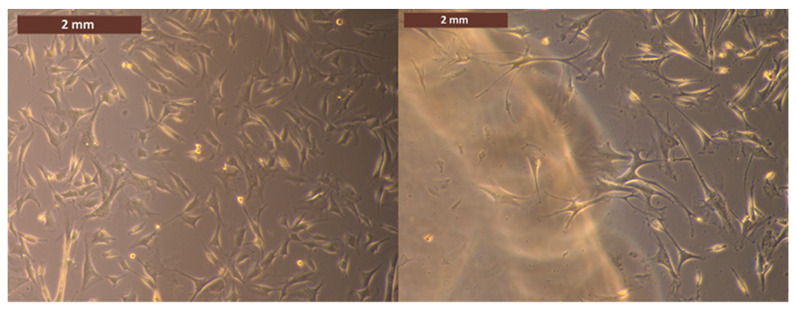
Cells in direct contact (48 h) with the vitamin B1 liner‘s aliquots (**right**) and without any sample—control (**left**). Image obtained with an inverted phase contrast microscope.

Microscopic images (Figure 8) of fibroblast cells incubated with the vitamin B1 liner material for 48 h showed very good biocompatibility of the material, with the slow diffusion of vitamin B1 or silicone fragments into the cell medium being nontoxic.

The vitamin B1 silicone dental lining material tested using MTT showed high cell viability and good biocompatibility for all incubation periods (24 to 120 h), highlighting that it is safe for clinical use. 

### 3.7. Bioadhesion and Mucoadhesion Tests

The tested liner materials showed a higher adhesion force on the oral mucosa tissue, especially for the vitamin-based materials (Table 5). The same samples revealed similar values when a synthetic cellulose membrane was used. A doubling of this value was observed for vitamin A lining material with a higher degree of swelling in artificial saliva (pH 6.8) after short periods of immersion in this medium. Lower values of adhesion force on oral mucosa were observed for the vitamin B1 liner, even if this sample had a higher value of water sorption capacity. Analysis of the adhesion values on the same substrates (Table 5) revealed that for all samples, the interaction with the biological medium was adequate. These values were three times higher for the vitamin-enriched liners compared with the reference. In this case, the degree of swelling of the materials followed by the release of small fragments of vitamins allowed the mucosal constituents to interact better with the materials in the consolidation stage of the adhesion process [31]. 

### 3.8. Histopathological Examination

**Figure 9:**
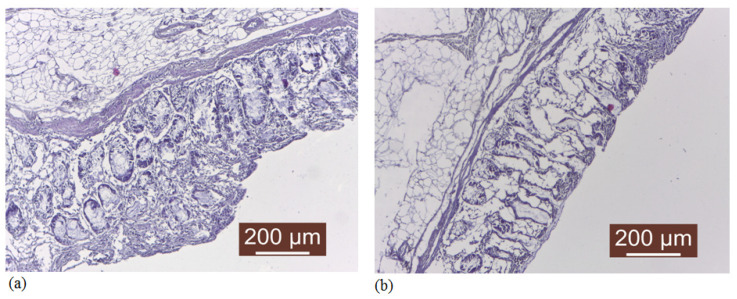
Normal autolyzed mucosa (**a**) and more pronounced autolysis phenomena in the presence of vitamin A liner (**b**).

Examination of the mucosa before and after exposure to vitamin-containing silicone liners indicated auto-inflammatory processes, especially by treatment with vitamin A liner, as can be seen in Figure 9. The main pathological disorders are related to more pronounced autolysis processes in the case of the vitamin A-containing liner. 

## 4. Discussion

The use of dental soft lining materials and silicone-based gels has often shown itself to be a good alternative to acrylic-based materials. Silicones are biocompatible and have good resistance to variable conditions, good shape and color stability [9], and can be used as long-term liners, often up to one year [10]. 

In this study, we aimed to improve the effectiveness of silicone-based lining materials by incorporation of the vitamins A, B1 and B6, which are involved in caries and periodontitis prevention and oral health. In general, there is little evidence to support the relation between vitamins and both gingival/periodontal disease and dental hard pathological processes, despite current literature suggesting that vitamins are helpful in the prevention and treatment of oral diseases. Future research on the effects of vitamins on the mouth, as well as specific studies of complex biological mechanisms, would have wider applications in dentistry and medicine [17].

The successful incorporation of vitamins into the silicone matrix was first evidenced using IR spectroscopy. The main absorption bands assigned to the silicone and the vitamins were found in the 1080–1012 cm^−1^ spectral range; 1258 and 789 cm^−1^ were specific to the Si-O-Si and Si-CH_3_ groups, respectively; and the specific absorptions of the vitamins were identified in the 1800–1300 cm^−1^ spectral range (Figure 2). The composition of lining materials via the incorporation of different plasticizers or fillers can increase their flexibility, mechanical strength and moisture resistance, with these being mainly responsible for the long-term lining material utilization [31,32].

The main role of denture lining materials is to improve the adaptability of the oral cavity without damaging the soft tissue. However, some disadvantages regarding the better fixation of the prosthesis could lead to poor functionality, which can be corrected by lining. One of the most desirable properties of dental lining is the tensile strength of materials, which particularly reflects the ability of a material to withstand failure during facial movements and especially during the insertion or removal of the prosthesis [33]. The highest values found for the reference lining material can be explained by the lower stress transmitted within the silicone matrix formed by compatible constituents (only base and catalyst), which prevents the development of cracking between the silicone chains. The decrease in tensile strength after the addition of vitamins during the synthesis process can be explained by an increase in the degree of crosslinking; the plasticizing effect of the vitamins; or the weak compatibility between the silicone matrix and this one, leading to a redistribution of the strain energy that led to higher localized stress, followed by an early fracture area. However, the addition of fillers can affect this behavior and then avoid the flow and orientation of polymeric chains, leading to the occurrence of multiple points of failure and, as a result, lower elongation [19]. The evolution of the strain–stress response of silicone liners subjected to 100% strain for ten loading–unloading cycles revealed a hysteretic response, with a stable maximum tensile stress, supporting the elastic properties of silicone liners, and thus, their high functional fatigue behavior.

The moisture behavior of the silicone-based lining materials was evaluated using the DVS technique, which determines the water vapor sorption capacity values from sorption/desorption isotherms at different relative humidity. The main factor that determines water adsorption is the composition of the materials, the presence of the pore or the hydrophilic domains in their structure that allow for water uptake (Table 3). It was found that the roughness of the surface can particularly lead to the accumulation of water and other biological fluids, deposition of salts and bacterial adhesion. These can affect the functionality of the oral tissues and, indirectly, the lining material’s longevity. An ideal roughness parameter of dental materials in clinical practice of 0.2 μm was established, above which an increase in bacterial retention is inevitable. In general, the silicone lining materials showed lower roughness than the corresponding acrylic ones. SEM images also revealed compact structures with the presence of small aggregates on the surface or of channels created during the crosslinking process (Figure 4).

The results of the DVS analysis showed that the hydrophobicity of the silicone lining materials was maintained after the vitamin incorporation, with sorption capacity values similar to the Mollosil reference sample and slightly higher in the case of the vitamin B1 and vitamin B6 liners due to the hydrosoluble behavior of the embedded vitamins (Table 3). The stability of the silicone-based liners was evaluated in artificial saliva (PBS pH 6.8), water and simulated biological media (PBS pH 7.4) by monitoring the degree of swelling gravimetrically and the structural changes using IR spectroscopy (Figure 6 and Figure 7). The vitamin A liner showed a constant swelling degree regardless of the swelling medium, while vitamin B1 and vitamin B6 liners showed higher swelling degrees, depending on the immersion medium. These samples doubled their swelling degrees after 48 h, especially in PBS pH 6.8 (Figure 5). The biological environment can be very aggressive due to the high chemical activity with large variations in different organs or tissues. These aspects are more pronounced in the oral cavity due to the large spectrum of mechanical forces, as well as the composition of the environment depending on the diet. On the other hand, once in the body, biomaterials should not degrade rapidly when they come into contact with physiological and pathological enzymes, as these need to have good stability and chemical resistance to the action of the environmental pH. Dental lining materials are also susceptible to changes during their application in the oral cavity. Materials containing additives or in situ produced ethanol or another plasticizer can leach out to produce a deterioration of the materials in terms of mechanical and physical properties (volumetric, hardening, color), leading to an increase in the cytotoxicity of the medium. 

During clinical use, soft lining materials show several disadvantages related to water absorption that usually leads to changes in the structure and properties, such as the loss of softness, distortion or surface damage. These are mainly influenced by the viscoelasticity and hydrophobicity and strongly affect long-term use, as the soft dental lining acts as a cushion for the oral mucosa, absorbing and redistributing the forces that occur in the stress-bearing edentulous areas [34]. When analyzing the structural changes using IR spectroscopy, poor stability of the reference lining material was observed. In the IR subtraction spectra of this sample, specific absorption bands assigned to the small silicone fragments were identified over 72 h. The IR subtraction spectra of vitamin-enriched lining materials also revealed the presence of a vitamin-specific band, showing their release in the simulated environment of the oral cavity (Figure 5 and Figure 6). Biocompatibility of dental lining material in the simulated biological medium of normal cell culture (Table 4) was achieved as a main step before the acceptance of these materials in clinical use, even though the in vitro tests of cytotoxicity could not fully reflect clinical conditions. Using a cell culture is a simple method of investigating cytotoxicity, especially as dental lining materials cause allergic reactions in contact with connective cells [35]. Given that the properties of the silicone matrix were modified by loading different lipo- and hydrosoluble vitamins in the crosslinking process and the resulting vitamin-containing liners exhibited very low water sorption capacity and swelling degrees with a small variation in the pH medium, it can be assumed that the mucoadhesive properties of these materials were somewhat of a disadvantage, indicating some instability for the lined prosthesis given the existence of friction and shear due to saliva flow, mastication and speech [36]. However, the values of the work of adhesion of the vitamin-lined materials with porcine oral mucosa showed slightly elevated values compared with the reference sample (Table 5). The effect of vitamins on the epithelial tissue of the oral cavity can be inhibitory, similar to growth factors or adrenergic agents. The use of vitamins as inhibitors was shown to be advantageous in oral pathologies caused by tumor proliferation. However, the local resistance of the oral mucosa was provided by saliva (rich in immunoglobulins); cellular fighters, namely, macrophage and lymphocytes; and rich vascular and lymphatic networks [37]. Oral mucosal modifications can be associated with systemic disease and particular conditions: auto-immune, skin disease, hematological, endocrine, metabolic or gastrointestinal disorders. Also, low levels of vitamins, such as vitamins A, B1, B6 or B12, are usually associated with recurrent aphthous ulcers. Thus, the oral mucosa is considered to be one of the most aggressive mucosas since it is exposed to various mechanical traumatic factors: thermal, pressure, chemical, irritant or infectious factors. On the other hand, the oral mucosa exhibited a permanent renewal and restoration feature, where the epithelial cells localized in the oral cavity were frequently replaced (every 14–21 days), ensuring a permanent turnover process.

## 5. Conclusions

Three vitamins (A, B1, B6) were for the first time encapsulated in soft Mollosil dental lining materials to improve their biocompatibility and mucoadhesiveness for better tolerance of prosthetic devices. The new composition of the resulting lining materials was analyzed using IR spectroscopy, highlighting the interaction of vitamins with the silicone matrix. The addition of vitamins was found to have a beneficial effect that was mainly manifested as improved biocompatibility, hydrolytic stability and mucoadhesiveness with the oral mucosa, and less as mechanical strength. The obtained lining materials showed good resistance in simulated biological environments but caused a pronounced autolysis phenomenon, as revealed by histopathological examination, supporting the fact that these materials have far-reaching implications in the treatment of oral diseases.

## Figures and Tables

**Table 1:** The composition of the prepared vitamin-containing silicone lining materials.

Prepared Samples	Mollosil Base, g	Mollosil Catalyst, g	Vitamin	Chloroform, mL
Mollosil (reference sample)	0.25	0.25	-	0.5
Vitamin A liner	0.25	0.25	1%	0.5
Vitamin B1 liner	0.25	0.25	1%	0.5
Vitamin B6 liner	0.25	0.25	1%	0.5

**Table 2:** Mechanical data of the Mollosil-embedded vitamin materials.

Sample	Sm ^a^, %	Tnm ^b^, MPa	Y ^c^, MPa
Mollosil reference sample	491	3.46	0.85
Vitamin A liner	292	0.89	0.38
Vitamin B1 liner	361	2.05	0.76
Vitamin B6 liner	277	1.28	0.69

^a^ Elongation at break; ^b^ stress at break; ^c^ Young’s modulus (calculated at 10% elongation).

**Table 3:** Surface parameters evaluated based on adsorption/desorption isotherms.

Surface	W (%)	$r_{pm}$ (nm)	BET Data *	
			Area (m^2^/g)	Monolayer (g/g) × 10^−2^
Mollosil reference sample	0.65	2.18	5.96	0.16
Vitamin B6 liner	0.65	2.82	4.60	0.13
Vitamin B1 liner	4.85	7.11	13.64	0.39
Vitamin A liner	1.88	1.89	19.82	0.56

W—water vapor sorption capacity; $r_{pm}$—average pore size; * BET data determined based on desorption branch of the isotherm (registered up to a relative humidity of 40%).

**Table 4:** MTT assay results of the vitamin B1 liner material.

Time	Control	Final Concentration
24 h	100.00 ± 16.17	100.43 ± 3.93
48 h	100.00 ± 14.14	95.38 ± 4.89
120 h	100.00 ± 10.77	95.15 ± 13.06

**Table 5:** The results of the bio- and mucoadhesion analysis of the silicone-based liner materials.

Sample	Bioadhesion		Mucoadhesion	
	Adhesion Force (N)	Work of Adhesion (mJ)	Adhesion Force (N)	Work of Adhesion (mJ)
Mollosil reference sample	0.06799 ± 0.00056	0.06407 ± 0.00301	0.00413 ± 0.00032	0.00613 ± 0.00101
Vitamin A liner	0.06178 ± 0.00294	0.09872 ± 0.00982	0.00713 ± 0.00110	0.01583 ± 0.00167
Vitamin B1 liner	0.06113 ± 0.00150	0.08695 ± 0.00787	0.00530 ± 0.00011	0.01450 ± 0.00091
Vitamin B6 liner	0.06146 ± 0.00283	0.07616 ± 0.000566	0.00527 ± 0.00011	0.01601 ± 0.00096

## Data Availability

Not applicable.
